# Emotion and attention interaction: a trade-off between stimuli relevance, motivation and individual differences

**DOI:** 10.3389/fnhum.2013.00364

**Published:** 2013-07-12

**Authors:** Leticia Oliveira, Izabela Mocaiber, Isabel A. David, Fátima Erthal, Eliane Volchan, Mirtes G. Pereira

**Affiliations:** ^1^Instituto Biomédico, Universidade Federal FluminenseNiterói, Brazil; ^2^Polo Universitário de Rio das Ostras, Universidade Federal FluminenseNiterói, Brazil; ^3^Institute of Biophysics Carlos Chagas Filho, Universidade Federal do Rio de JaneiroRio de Janeiro, Brazil

**Keywords:** attention, emotion, individual differences, stimuli relevance, motivation

## Abstract

Mounting evidence suggests that the neural processing of emotional stimuli is prioritized. However, whether the processing of emotional stimuli is dependent on attention remains debatable. Several studies have investigated this issue by testing the capacity of emotional distracters to divert processing resources from an attentional main task. The attentional load theory postulates that the perceptual load of the main task determines the selective processing of the distracter. Although we agree with this theory, we also suggest that other factors could be important in determining the association between the load of the main task and distracter processing, namely, (1) the relevance of the to-be ignored stimuli and (2) the engagement in the main task due to motivation. We postulate that these factors function as opposite forces to influence distracter processing. In addition, we propose that this trade-off is modulated by individual differences. In summary, we suggest that the relationship between emotion and attention is flexible rather than rigid and depends on several factors. Considering this perspective may help us to understand the divergence in the results described by several studies in this field.

## Introduction

A large number of studies has suggested that the processing of emotion-laden stimuli is prioritized because of their relevance to survival (Ohman et al., 2001; Vuilleumier et al., 2001; Anderson et al., 2003; Phelps et al., 2006). The experimental paradigms used to demonstrate this prioritization include detection, search, interference, masking, and the attentional blink. Many studies have interpreted the interference produced by emotional distracters as evidence that their processing is not only prioritized, but that it occurs in an obligatory fashion that is independent of attention (Ohman et al., 2001; Vuilleumier et al., 2001). Consistent with this view, neuroimaging studies have reported that amygdala responsivity to emotional stimuli is not modulated by attentional manipulations, supporting the idea that emotional stimuli are processed “automatically” (Vuilleumier et al., 2001; Dolan and Vuilleumier, 2003).

However, it is also known that visual processing capacity is limited. According to Lavie's theory (Lavie, 2005), the perceptual load of relevant information determines the selective processing of irrelevant information. For instance, a high perceptual load situation that engages full capacity levels to process task-relevant stimuli would leave no spare capacity to perceive task-irrelevant stimuli. However, in a low perceptual load situation, any capacity not taken up by the perception of task-relevant stimuli would involuntarily “spill over” to the perception of task-irrelevant distracters. This hypothesis has been investigated by the manipulation of the attentional load of the main task, sparing different levels of brain resources to process an emotional distracter. According to the automaticity premise, if emotional stimuli are processed automatically, then this processing should occur even when brain resources are fully consumed by the main task. However, a number of neuroimaging and behavioral studies support an alternative hypothesis that emotional stimuli compete for neural representation, thus requiring attentional resources. These studies employed highly demanding tasks while presenting emotional distracters and did not show any evidence of the differential processing of emotional stimuli, suggesting that the perception of emotional stimuli is dependent on attention (Pessoa et al., 2002a,b, 2005; Mitchell et al., 2007; Pourtois et al., 2010). These results have emphasized a reliance on attentional resources in processing emotional stimuli. This “attentional load concept” appears to be a reasonable explanation for the discrepancies that exist between studies that suggest the automaticity of emotional stimuli processing (Vuilleumier et al., 2001; Anderson et al., 2003) and those that support attentional dependence for emotional processing (Pessoa et al., 2002a,b; Silvert et al., 2007). However, the attentional load concept does not fully explain the divergent results reported in the literature (Muller et al., 2008; Fenker et al., 2010) and it may be more flexible than initially thought. Then, the way in which the processing of emotional stimuli depends on attention remains unclear. In this brief review, we present factors other than the perceptual load of task-relevant processing that might be responsible for a greater or lesser degree of the processing of task-irrelevant emotional stimuli.

## Factors that determine the availability (and allocation) of processing resources to emotional stimuli

**Figure 1:**
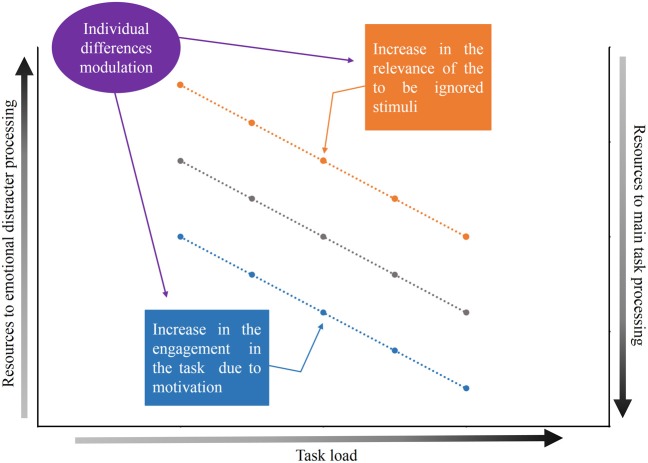
**Graphic representation of the theoretical proposal**. The gray line represents the relationship between the load of the main task, the allocation of resources for the processing of the emotional distracters and the allocation of resources for the processing of the main task. The resources available for processing of the distracters is expected to diminish (or even be abolished) as the load of the main task increases. The blue line represents the modulation caused by the increase in the engagement in the task due to motivation and the orange line represents the modulation by the increase in the relevance of the to-be ignored stimuli. Both the engagement in the main task due to motivation and the relevance of the to-be ignored stimuli may vary from individual to individual.

The first step in understanding the factors that define the extent to which emotional stimuli are processed when they are presented as distracters involves the participants ability to concentrate on the relevant task and ignore the emotional stimuli. In this situation, two factors are fundamental in determining the amount of processing resources that are allocated to emotional stimuli: (1) the relevance of the to-be ignored stimuli (the distracter) and (2) the engagement in the main task due to motivation. Possibly, there is a trade-off between these two factors, and the combination of these factors determines the availability of processing resources to emotional stimuli. Here, we propose that emotional distracter processing is dependent on the main task load (Pessoa et al., 2002a,b; Erthal et al., 2005), but that this association might be modulated by the relevance of the distracter and the engagement in the main task due to motivation. Specifically, we propose that an enhanced relevance of the distracter might facilitate its processing and an increase in the load of the relevant task would be necessary to eliminate the processing of the distracter. However, an increase in the engagement in the main task due to motivation would diminish (and potentially eliminate) the processing of the distracter, even if the distracter is an emotional stimulus. We also suggest that differences between individuals may modulate the trade-off between the relevance of the distracters and the engagement to perform the main task due to motivation (see Figure 1). In the next sections, we will discuss these factors in greater detail.

## Relevance of the to-be ignored stimuli

The relevance of the to-be ignored stimuli may be a decisive factor in whether they will be processed in a privileged way by the brain. Stimulus appraisal determines the extent to which stimuli will be considered relevant to the individual's goals or well-being, which in turn dictates subsequent attention allocation (Sander et al., 2005). Recently, Purkis et al. (2011) reported that strong interference was produced in a visual search task involving pictures related to the television program “Dr Who” performed by fans of this program (Purkis et al., 2011). Moreover, in the same task, interference generated by pictures of spiders was strongly correlated with scores on a spider fear questionnaire, whereas interference generated by pictures related to Dr Who was correlated with scores on a Dr Who expertise questionnaire. This result suggested that the magnitude of the attentional bias for positive and negative emotional stimuli was dependent on the relevance of these stimuli for each individual. Consistent with this finding, a person's own name has been shown to modulate the attentional blink (Shapiro et al., 1997) and can be detected substantially faster than control names in a visual search task (Mack and Rock, 1998). In previous studies, emotional stimuli acquired relevance via learning mechanisms, but their importance might also be biologically determined. Brosch et al. (2008) examined the Event-Related Potential (ERP) latency of participants who performed a dot-probe detection task when biologically relevant stimuli (pictures of angry faces or babies) served as cues (Brosch et al., 2008). This study found that both emotional stimuli were able to capture attention within a similar time course, resulting in the conclusion that biologically relevant stimuli, whether fear- or nurture-relevant, have access to preferential processing (see also Brosch et al., 2007). Furthermore, recent studies using highly relevant emotional stimuli in phobic participants suggested that the relevance of the stimuli determined their ability to obtain brain resources even when these brain resources were very limited. For instance, fear-related stimuli interfered with the performance of participants who were highly fearful of snakes or spiders, even when their attention was directed elsewhere or when attentional resources were limited (Okon-Singer et al., 2011). In addition (Norberg et al., 2010) showed that spider-fearful participants demonstrated greater late-positive potentials (LPPs) toward spiders than toward mushrooms even while performing a highly demanding discrimination task (Norberg et al., 2010).

Taken together, these findings suggested that the relevance of a stimulus determines its subsequent attention allocation. Interestingly, these findings also suggested that stimulus relevance may be enhanced if the stimulus is personally meaningful. Thus, stimuli processing is prioritized and might recruit additional resources even in situations where visual information attentional resources are very limited.

## Engagement in the main task—the effects of motivation

As previously discussed, the relevance of the to-be ignored stimuli may be a key factor to determine the processing of these stimuli. On the other hand, the engagement in the main task may counteract the disruptive effect of the distractor, particularly in situations in which the main task is very demanding (Lavie and de Fockert, 2003; Lavie, 2005). However, the demand of the main task itself is not sufficient to determine the brain resources allocation to target and prevent the distractor processing (see Lavie and de Fockert, 2003). In this review, we will discuss the hypothesis that another key factor is the motivation of the participants in performing the main task. We hypothesize that the increase in this motivation produces an increase in the allocation of resources, which in turn, enhances the efficiency of performing the main task, resulting in diminished processing of the distracter (Figure 1). In other words, engagement in the main task due to motivation might allow the participants to upregulate top–down control processes that bias the selection of the task information, thereby leading to more efficient task processing and reducing the processing of the distracter. Recent studies have shown that motivation produces an enhancement in executive function (Small et al., 2003, 2005; Engelmann and Pessoa, 2007; Mohanty et al., 2008; Della and Chelazzi, 2009; Engelmann et al., 2009; Padmala and Pessoa, 2011). Motivation can enhance detection sensitivity during a challenging attention task, and visual sensitivity is increased as a function of absolute monetary incentive value (Engelmann and Pessoa, 2007). Furthermore, Della and Chelazzi (2009) showed that participants became more efficient at selecting targets that were consistently associated with high-magnitude rewards (Della and Chelazzi, 2009).

More importantly, Padmala and Pessoa (2011) showed that motivation enhances the efficiency of performing a main task and simultaneously ignoring the distracters in a conflict task (Padmala and Pessoa, 2011). Specifically, the participants performed a response conflict task (a picture of a house or building was presented with an irrelevant congruent/neutral/incongruent word) under two motivational contexts: (1) during the reward condition - a cue stimulus of “$20” signaled that the participants would be rewarded for fast and correct performance and (2) during the no reward condition - a cue stimulus of “$00” signaled that no reward was involved. Behavioral results also showed that the reward decreased both the interference (incongruent vs. neutral) and facilitation effects (congruent vs. neutral), which was consistent with the notion that motivation for the main task enhanced attentional filtering, thereby reducing the influence of the task-irrelevant word item. Furthermore, right intraparietal sulcus activation was observed in conjunction with decreased word-related responses in the left fusiform gyrus in the reward condition compared with that observed in the no-reward condition. This is consistent with the idea that enhanced attentional control elicited by a reward biased the processing in a way that decreased the influence of irrelevant word stimuli.

Taken together, the results of these studies are consistent with a model in which motivation applied to the main task functions to upregulate top–down control processes leading to more efficient task-requirements, thereby helping to diminish distracter processing. In other words, engagement in the main task due to motivation induces specific effects in the attentional network that are related to increased efficiency in the ability to ignore distracters, even when participants perform a relatively low-load task (see Figure 1).

## Individual differences: the effects of personality traits

In this section, we add another element that reveals that the relationship between emotion and attention is flexible rather than rigid. We will argue that differences between individuals modulate the availability (and allocation) of processing resources to emotional stimuli as a function of personality traits. Both (a) the *relevance of the to-be ignored stimuli* and (b) *engagement to perform the main task due to motivation* could vary from individual to individual.

Regarding the relevance of the to-be ignored stimuli, mounting evidence suggests that individual differences are important predictors of sensitivity to emotional stimuli and help to explain the variable effects of emotional stimuli. For example, studies within the literature on anxiety have revealed that anxious participants exhibit greater interference from threat-related stimuli. MacLeod et al. (1986) showed that highly anxious participants are faster to detect neutral targets that are presented spatially close to threat words. It has also been shown that they have larger late positive potentials (LPP)—an index of emotional processing—to distractive unpleasant pictures compared to low anxiety subjects (Mocaiber et al., 2009). Pérez-Dueñas et al. (2009) showed that individuals with high trait anxiety did not present the expected inhibition of return effect when threatening stimuli was the target in a standard spatial cueing procedure. This result supports the hypothesis of increased attentional capture by negative stimuli, in high trait anxiety individuals. More recent studies have also investigated the extent to which amygdala responses to threat-related distracters depend upon individual anxiety levels (Bishop et al., 2004). Whereas low-anxiety individuals only showed increased amygdala responses to attended fearful faces, highly anxious individuals showed increased amygdala responses to both attended and unattended threat-related stimuli. These findings suggest that the threat value of a stimulus varies as a function of a participant's anxiety level, and consequently, affect how meaningfully this stimulus is considered. However, other studies have suggested that attention is important even for highly anxious individuals (Fox et al., 2005; Bishop et al., 2007). For example, Bishop et al. (2007) found a positive correlation between the anxiety state and amygdala reactivity to threat-related distracters under low, but not high, attentional load. In other words, even when a stimulus was personally relevant, its processing was not immune to attention availability. Thus, these studies suggest that specific individual sensitivity to a stimulus might define the distribution of brain resources.

Studies exploring affective style also provides evidences that individual differences are important predictors of sensitivity to emotional stimuli. For instance, high neuroticism predicts greater amygdala activation to a task involving emotional processing (Haas et al., 2007). Oliveira et al. (2009) demonstrated that high positive affect trait individuals showed attenuated autonomic reactions to the mutilation pictures. Furthermore, Souza et al. (2007) showed that individual predispositions, e.g. negative affective trait and vagal tone, influence heart period recovery after a speech stress task.

Individual differences appear to be important not only regarding the relevance of the to be ignored stimuli but also regarding the engagement in the main task due to motivation. Personality traits can influence how the participants react to incentives during a relevant task (Locke and Braver, 2008; Engelmann et al., 2009; Savine et al., 2010). For instance, Locke and Braver (2008) showed that motivational incentives were associated with improved performance and greater cognitive control and that the relationship between reward and brain activity may be modulated by how participants perceive a reward (Locke and Braver, 2008). Moreover, Padmala and Pessoa (2011) found that the functional connectivity between the right intraparietal sulcus and the right nucleus accumbens is also correlated with the behavioral activation system (BAS) scores, which suggests that reward sensitivity influences how the regions interact with each other during the performance of a highly motivating task (Padmala and Pessoa, 2011).

In summary, the trade-off between engagement in the main task and the relevance of the to-be ignored stimuli appears to be modulated by differences between individuals. As illustrated in Figure 1, we suggest that the amount of shifting produced by the relevance of a distracter and the engagement in the main task due to motivation is dependent on individual differences.

## Concluding remarks

Currently, the literature presents two opposing views on how emotion and attention interact: the classical view, which states that emotional stimuli are processed in an automatic fashion due to its relevance for survival (Vuilleumier et al., 2001; Anderson et al., 2003) and a competing view, which states that even emotional stimuli or events are regulated by top-down influences and are not immune to attention availability (Pessoa et al., 2002a,b, 2005; Erthal et al., 2005). However, it appears that these mechanisms are not mutually exclusive, as was initially proposed.

Here, we discussed how emotion and selective attention interact with each other. Despite Lavie's theory, which postulates that the perceptual load of relevant information determines the selective processing of irrelevant information, debate is ongoing regarding the dependency of attention to emotional processing. Although we are in agreement with Lavie's theory, we suggest other important key issues in determining the association between the load of the main task and distracter processing: (1) the relevance of the to-be ignored stimuli and (2) engagement in the main task due to motivation. We postulate that these factors function as opposing forces to influence distracter processing. Specifically, the relevance of the distracter facilitates its brain processing and increases the effect of these stimuli on behavior. In contrast, increased motivation to perform the main task increases the engagement of the participant and reduces the resources needed to process the affective distracter. Furthermore, it is important to note that both the relevance of the stimulus and the engagement in the main task due to motivation are modulated by individual differences. We propose that there is a trade-off between these two aspects and that the combination of these factors could generate different outputs. For example, the relevance of the stimuli does not ensure that these stimuli will be necessarily processed. As previously stated, the participant's own names appear to have facilitated brain processing, although evidence also suggests that this facilitation is subject to capacity limitations (Harris et al., 2004). Thus, the trade-off between these factors will help to determine whether the emotional distracter can be processed in a privileged way. In summary, we suggest that emotional distracter processing is dependent on a balance between the relevance of the distracter and the engagement in the main task due to motivation. As illustrated in Figure 1, we also suggest that this trade-off is modulated by individual differences. Thus, the relationship between emotion and attention is flexible rather than rigid and is dependent on several aspects. Therefore, considering this perspective may help in understanding the divergence described by several studies in this field.

### Conflict of interest statement

The authors declare that the research was conducted in the absence of any commercial or financial relationships that could be construed as a potential conflict of interest.
